# Knee Arthroplasty Types: An Educational Framework for Multidisciplinary Healthcare Teams

**DOI:** 10.7759/cureus.89127

**Published:** 2025-07-31

**Authors:** Uday Mahajan, Meraj Akhtar, Baijaeek Sain, Arnab Sain

**Affiliations:** 1 Trauma and Orthopaedics, Queen Elizabeth Hospital Birmingham, Birmingham, GBR; 2 Trauma and Orthopaedics, Lincoln County Hospital, Lincoln, GBR; 3 Trauma and Orthopaedics, Royal Berkshire NHS Foundation Trust, Reading, GBR; 4 Trauma and Orthopaedics, Worthing Hospital, Worthing, GBR

**Keywords:** knee arthroplasty, multidisciplinary education, partial knee replacement, revision total joint arthroplasty, total knee replacement (tkr)

## Abstract

Knee arthroplasty is a widely performed surgical procedure that significantly improves the quality of life for patients with advanced knee joint diseases. However, many multidisciplinary healthcare professionals lack a clear understanding of the types and subtypes of knee arthroplasty, which is essential for effective perioperative care and rehabilitation planning. This narrative review categorises knee arthroplasty into three main types: total knee arthroplasty, partial knee arthroplasty, and revision knee arthroplasty. Total knee arthroplasty remains the most common procedure, with subtypes based on implant constraint, fixation method, bearing surface, alignment philosophy, surgical approach, and technological aids. Partial knee arthroplasty includes unicompartmental and patellofemoral replacements, offering faster recovery and more natural knee kinematics in appropriately selected patients, although with higher revision rates. Revision knee arthroplasty addresses failed implants using one-stage or two-stage procedures with specialised components such as constrained condylar and hinged implants, stem extensions, augments, and megaprostheses, but carries higher surgical complexity and complication risks. This educational framework aims to enhance multidisciplinary understanding of knee arthroplasty procedures, supporting better communication, patient education, and rehabilitation planning. Future research should validate its impact in educational settings and explore ongoing innovations in implant designs and surgical technologies to optimise patient outcomes.

## Introduction and background

Knee arthroplasty is a widely performed and continually evolving surgical procedure that has significantly improved the quality of life for patients with debilitating knee joint diseases, such as advanced osteoarthritis and rheumatoid arthritis [1]. As global life expectancy increases and the prevalence of degenerative joint diseases rises, the global demand for knee arthroplasty is projected to grow substantially driven by epidemiological trends such as aging populations, rising obesity rates, and heightened expectations for functional mobility in later life [2].

This increase in surgical volume highlights the critical need for a comprehensive understanding of knee arthroplasty among multidisciplinary healthcare teams, including junior doctors, nurses, physiotherapists, and allied health professionals, who play vital roles in perioperative management and rehabilitation [3]. Effective postoperative recovery requires coordinated care encompassing pain management, mobilisation, patient education, and monitoring for complications such as infection, thromboembolism, and joint stiffness [3].

The increasing prevalence of knee osteoarthritis has led to the development of a diverse array of prosthetic implant designs and surgical techniques, necessitating a structured framework for classification and comprehension [4]. Variations exist in implant constraint levels, fixation methods, bearing surface materials, alignment philosophies, surgical exposure approaches, and technological aids. These choices are tailored to patient-specific factors such as bone quality, ligament integrity, and deformity severity to optimise functional outcomes, implant longevity, and patient satisfaction [4].

Given the potential for ambiguity in communication among surgeons and allied health professionals, a standardized classification system is imperative for ensuring patient safety, maintaining accurate clinical records, and facilitating clear communication [5]. Standardisation also plays a role in appropriate remuneration processes, robust data collection for joint registries, and evaluation of long-term surgical outcomes [5].

Despite its clinical importance, there is currently a lack of integrated educational frameworks that summarise knee arthroplasty types and subtypes in a way that is accessible to non-orthopaedic healthcare staff. This knowledge gap can limit the ability of multidisciplinary teams to anticipate postoperative care needs, support rehabilitation protocols, and effectively educate patients about their surgical procedures and recovery pathways.

This narrative review aims to provide a clear and structured overview of the main types of knee arthroplasty, namely, total knee arthroplasty (TKA), partial knee arthroplasty (PKA), and revision knee arthroplasty, highlighting their classifications, design philosophies, and clinical relevance to enhance multidisciplinary team understanding and improve patient care outcomes.

## Review

This narrative review was conducted with the objective of presenting a structured overview of the different types of knee arthroplasty, namely, total, partial, and revision, to support multidisciplinary healthcare teams involved in the care of patients undergoing these procedures. The rationale for this review is rooted in the increasing demand for knee arthroplasty globally, driven by demographic shifts such as population aging and rising osteoarthritis prevalence. To identify relevant literature, a non-systematic search was carried out using the PubMed, Google Scholar, and Scopus databases covering the period from January 2000 to May 2024. Search terms included combinations of “knee arthroplasty”, “total knee replacement”, “partial knee replacement”, “revision knee arthroplasty”, “implant constraint”, “prosthesis types”, “alignment philosophy”, “robotic knee surgery”, and “multidisciplinary care”.

The review included English-language peer-reviewed articles comprising clinical studies, narrative and systematic reviews, and research that explored classifications, surgical techniques, implant types, and alignment philosophies relevant to knee arthroplasty. Articles such as isolated case reports, editorial pieces, or those lacking relevance to surgical classification or clinical applicability were excluded. Selected articles were first screened by title and abstract, followed by full-text evaluation to confirm relevance and quality. Data were extracted and organized thematically to support a comprehensive yet accessible narrative structure. The analysis focused on six main domains within total knee arthroplasty, namely, implant constraint, fixation, bearing surface, alignment strategy, surgical approach, and technological integration, while also addressing the classifications, indications, and limitations specific to partial and revision knee arthroplasty. The aim was to synthesise current knowledge in a way that is clinically relevant, educationally useful, and easily understandable by diverse healthcare professionals.

Overview of knee arthroplasty types

Knee arthroplasty can be broadly classified into three main types: TKA, PKA, and revision knee arthroplasty [4]. Each type addresses specific pathological conditions and patient needs, and within each, further subtypes exist based on implant design, fixation method, alignment philosophy, and technological adjuncts. Understanding this classification framework enables multidisciplinary teams to grasp surgical goals, postoperative considerations, and rehabilitation implications relevant to their clinical practice. The following sections provide a structured overview of each type and its subtypes.

Total Knee Arthroplasty

TKA is the most common type of knee replacement surgery performed worldwide [4]. It is indicated for patients with severe degenerative joint diseases affecting multiple compartments of the knee, such as osteoarthritis, rheumatoid arthritis, or post-traumatic arthritis [6]. The procedure involves resurfacing the distal femur, proximal tibia, and often the patella with prosthetic components to restore joint alignment, stability, and function. TKA aims to relieve pain, correct deformity, and improve mobility, thereby enhancing patients’ overall quality of life.

The subtypes of TKA are presented below*.*

Implant constraint: Implant constraint refers to the level of stability a knee replacement implant provides, which depends on the condition of the patient’s ligaments and overall joint stability. In clinical practice, the choice of implant is based on the patient's anatomy, the extent of joint damage, and ligament integrity. Cruciate-retaining (CR) implants are typically selected for patients whose posterior cruciate ligament (PCL) is intact and functioning, as this allows preservation of more natural knee movement. Posterior-stabilised (PS) implants, which include a built-in cam-and-post mechanism to mimic the stabilising role of the PCL, are used when the ligament is deficient or removed. When there is moderate ligament laxity or deformity, surgeons may opt for a condylar-constrained knee (CCK) implant, which offers greater stability in both the front-to-back and side-to-side planes. In more complex cases, such as those involving severe instability, neuromuscular disease, or significant bone loss, hinged implants are used. These function like a mechanical hinge and are considered a last resort due to their increased constraint. Ultimately, the selection is based on preoperative assessment and intraoperative findings, balancing joint stability with the patient’s expected function and activity level [7].

Fixation: Fixation describes how the implant is attached to bone. Cemented fixation uses polymethylmethacrylate bone cement to secure the implant components, providing immediate stability and is commonly used in elderly patients with osteoporotic bone. Uncemented fixation relies on bone ingrowth into porous-coated implant surfaces for biological fixation, making it preferable for younger, more active patients with good bone quality. Hybrid fixation combines cemented fixation of one component, usually the tibial component, with uncemented fixation of the other, usually the femoral component, to optimise both initial stability and long-term bone ingrowth. While cemented fixation remains the gold standard, particularly in older patients with lower bone quality, studies have not consistently demonstrated superior long-term survivorship with uncemented or hybrid technique [8].

Bearing surface: The bearing surface refers to the material interface between the femoral and tibial components and significantly impacts wear characteristics and implant longevity. Metal-backed tibial components are the most commonly used configuration, consisting of a metal tray with a polyethylene insert that balances durability with modularity. By contrast, all-polyethylene tibial components are composed entirely of polyethylene, presenting a simpler design that is often chosen for low-demand patients. Highly cross-linked polyethylene inserts provide enhanced wear resistance compared to conventional polyethylene, thereby reducing particle generation and the risk of osteolysis. In addition, fixed-bearing designs have the polyethylene insert securely attached to the tibial tray, whereas mobile-bearing designs allow rotation or translation of the polyethylene insert to distribute loads more evenly and mimic natural knee kinematics.However, mobile-bearing designs are mechanically more complex and may carry a slightly higher risk of mechanical complications. Moreover, studies have not demonstrated consistent advantages in functional outcomes or implant survivorship compared to fixed-bearing designs [9].

Alignment philosophy: Alignment philosophy guides the surgical technique to restore optimal knee alignment. Mechanical alignment positions the implant components perpendicular to the mechanical axis of the limb to achieve neutral alignment, a method historically considered the gold standard for implant longevity. Kinematic alignment aims to restore the patient's pre-arthritic joint lines and knee kinematics, resulting in a more natural feel and movement. In contrast, patient-specific alignment utilises imaging and custom instrumentation or implants that are tailored to each patient’s unique anatomy to optimise joint kinematics and implant positioning [10].

Surgical approaches: The choice of surgical approach significantly affects joint exposure, recovery, and rehabilitation outcomes. The medial parapatellar approach is the most commonly used technique, providing excellent joint exposure through a medial incision with patellar eversion. The subvastus approach preserves the quadriceps tendon attachment to the patella, which may reduce postoperative pain and facilitate faster functional recovery. The midvastus approach involves splitting the vastus medialis muscle, offering a balance between sufficient exposure and muscle preservation. Lastly, minimally invasive approaches utilise smaller incisions with specialised instruments to minimise soft tissue disruption, potentially enhancing early recovery, although these techniques require significant surgeon expertise to perform safely and effectively [11].

Technological advances: Technological advances in knee arthroplasty have significantly enhanced surgical accuracy and outcomes. Manual instrumentation relies on traditional jigs and anatomical landmarks, requiring substantial surgical experience to achieve accurate alignment. By contrast, computer navigation systems provide real-time intraoperative alignment data, improving precision and reducing the risk of alignment errors. Robotic-assisted surgery integrates preoperative planning with robotic guidance to achieve precise bone resection and implant placement, potentially enhancing functional outcomes and implant longevity by improving component positioning and joint balance [12].

In sum, understanding TKA subtypes, including implant constraint, fixation, bearing surfaces, alignment strategies, surgical approaches, and technological aids, enables multidisciplinary healthcare teams to anticipate postoperative needs, support optimal rehabilitation, and provide informed patient education, ultimately enhancing the quality of care delivered (see Table 1).

**Table 1:** Summary of total knee arthroplasty subtypes and classifications

Domain	Subtypes/options	Key features and clinical relevance
Implant constraint	- Cruciate-retaining (CR) - Posterior-stabilised (PS) - Condylar-constrained knee (CCK) - Hinged	CR preserves PCL for natural kinematics; PS substitutes PCL with cam-post mechanism; CCK provides added stability; hinged implants used in severe instability or bone loss [7].
Fixation	- Cemented - Uncemented - Hybrid	Cemented offers immediate fixation; uncemented relies on bone ingrowth; hybrid combines both for optimal stability and integration [8].
Bearing surface	- Metal-backed tibial component - All-polyethylene tibial component - Highly crosslinked polyethylene	Metal-backed is modular and durable; all-poly is simpler and cost-effective; highly crosslinked polyethylene reduces wear and osteolysis risk [9].
Alignment philosophy	- Mechanical alignment - Kinematic alignment - Patient-specific alignment	Mechanical aims for neutral axis; kinematic restores pre-arthritic anatomy; patient-specific customises implants based on imaging for improved kinematics [10].
Surgical approaches	- Medial parapatellar - Subvastus - Midvastus - Minimally invasive	Medial parapatellar is most common; subvastus and midvastus preserve quadriceps function; minimally invasive uses smaller incisions for faster recovery [11].
Technology	- Manual instrumentation - Computer navigation - Robotic-assisted surgery	Manual relies on surgeon expertise; computer navigation improves alignment accuracy; robotic-assisted offers precise bone resection and implant placement for better outcomes [12].

Partial Knee Arthroplasty

PKA is a surgical procedure that replaces only the diseased compartment(s) of the knee joint, preserving healthy bone, cartilage, and ligaments in the unaffected compartments. It is indicated for patients with osteoarthritis confined to a single compartment (medial, lateral, or patellofemoral), aiming to relieve pain and restore function while maintaining more natural knee kinematics [5].

Compared to TKA, PKA involves smaller incisions, less bone resection, and reduced soft tissue disruption, resulting in faster postoperative recovery, reduced pain, and a more physiological feel during movement [1]. However, its success relies on careful patient selection and precise surgical technique.

The subtypes of PKA are presented below. 

Unicompartmental knee arthroplasty (UKA): UKA involves replacing either the medial or lateral compartment of the knee joint. Medial UKA is the most common form and is indicated for patients with isolated medial compartment osteoarthritis who have intact ligaments and minimal deformity. By contrast, lateral UKA is less commonly performed due to different anatomical and loading characteristics of the lateral compartment, but it remains indicated for patients with isolated lateral compartment disease.

The advantages of UKA include preservation of both cruciate ligaments, which maintain normal knee kinematics and proprioception. Patients often experience quicker rehabilitation, greater range of motion, and higher satisfaction compared to TKA when appropriately selected [5].

Patellofemoral arthroplasty (PFA): PFA is performed in patients with isolated patellofemoral joint arthritis that has not responded to conservative management. It involves resurfacing the patella and the trochlear groove of the femur [13]. The advantages of PFA include a less invasive procedure with preservation of tibiofemoral compartments, leading to a faster recovery and more natural knee function. However, patients may later develop arthritis in other compartments, necessitating conversion to TKA, which generally remains feasible with standard components and instrumentation [13].

While PKA offers numerous benefits, it also has certain limitations. One major limitation is its higher revision rate compared to TKA, primarily due to the progression of osteoarthritis in the untreated compartments or implant failure over time. In addition, PKA is technically demanding, requiring accurate implant positioning and meticulous patient selection to avoid complications such as instability, bearing dislocation, or persistent residual pain. Careful preoperative evaluation of ligament integrity, deformity correction requirements, and overall knee alignment is crucial to ensure optimal outcomes [14].

In sum, PKA provides a bone-preserving, kinematics-friendly alternative to TKA for selected patients. Understanding its indications, subtypes, and limitations enables multidisciplinary teams to support postoperative care, rehabilitation planning, and patient education effectively [13] (see Table 2).

**Table 2:** Summary of partial knee arthroplasty subtypes and classifications

Domain	Subtypes/options	Key features and clinical relevance
PKA type	Unicompartmental knee arthroplasty (UKA)	- Replaces medial or lateral compartment only. - Preserves both cruciate ligaments for natural kinematics. - Faster recovery and greater range of motion compared to TKA [14].
PKA type	Patellofemoral arthroplasty (PFA)	- Replaces patella and trochlear groove only. - Indicated for isolated patellofemoral arthritis. - Preserves tibiofemoral compartments for more natural knee function [5,13].
Advantages	- Bone and ligament preservation - Faster rehabilitation - More physiological feel	PKA procedures result in smaller incisions, less soft tissue disruption, reduced postoperative pain, and quicker return to function [5,13].
Limitations	- Higher revision rates - Technical demands	Progression of arthritis in other compartments may require conversion to TKA in future; surgical precision and patient selection are critical for success [13].

Revision Knee Arthroplasty

Revision knee arthroplasty involves the replacement of a failed primary or previous knee arthroplasty to restore function, relieve pain, and improve quality of life. It is a more complex procedure than primary knee replacement due to altered anatomy, bone loss, ligamentous insufficiency, and higher complication risks [11].

The most common indications for revision knee arthroplasty include aseptic loosening, infection, instability, wear-related osteolysis, periprosthetic fracture, and arthrofibrosis. Failures may be broadly classified as early (within two years of the index procedure), often related to infection, instability, or surgical technique, and late (beyond two years), more commonly due to aseptic loosening, wear, or osteolysis. Recognising this distinction can aid clinical reasoning and help guide appropriate investigations, surgical planning, and inter-professional management [15,16].

The subtypes of revision knee arthroplasty are presented below. 

One-stage revision: It involves removing the failed components and implanting new prostheses within the same surgical procedure. This approach is primarily used in cases of aseptic loosening or infections caused by low-virulence organisms where thorough debridement and immediate reimplantation are feasible [17].

Two-stage revision: It is considered the gold standard for managing periprosthetic joint infections [17]. This strategy involves two distinct procedures. The first stage includes removal of the infected implants, extensive surgical debridement, and placement of an antibiotic-loaded cement spacer to control the infection. After infection eradication, which typically requires a period of six to twelve weeks, the second stage involves reimplantation of new prosthetic components. Although more resource-intensive than single-stage revision, the two-stage approach has demonstrated higher infection eradication rates and remains the preferred treatment modality for chronic or complex infections.

Due to bone loss and ligament insufficiency commonly encountered in revision knee arthroplasty, specialised implants and adjuncts are often required to achieve adequate stability and function [18]. Constrained condylar knee (CCK) implants provide increased stability while allowing some degree of rotational freedom and are used when collateral ligaments are deficient but a fully hinged construct is not necessary. Hinged implants are fully constrained designs that act as a mechanical hinge between the femur and tibia and are indicated in cases of severe instability, extensive bone loss, or after tumour resections. Stem extensions consist of long stems attached to femoral or tibial components to bypass areas of bone loss and transfer mechanical loads to the diaphyseal bone, enhancing implant fixation and stability. Augments, such as metal wedges or blocks, are utilised to fill bone defects and restore joint line height, particularly in defects of the tibial plateau or distal femur. In more severe cases, porous metaphyseal sleeves or tantalum cones may be used to achieve stable fixation within the metaphyseal bone and promote osseointegration where standard augments are insufficient. Megaprostheses are used in extreme bone loss scenarios where standard revision implants are inadequate, such as in oncological reconstructions or massive failed revision cases, providing structural replacement for extensive bone and soft tissue loss [18].

Revision knee arthroplasty is associated with several important clinical considerations [4]. These procedures typically involve longer operative times and greater intraoperative blood loss compared to primary TKA. Patients undergoing revision surgery face increased risks of complications, including infection, stiffness, neurovascular injury, and thromboembolic events. Furthermore, although functional outcomes following revision TKA are generally lower compared to primary procedures, many patients still achieve meaningful improvements in pain relief and mobility, which can significantly enhance their overall quality of life.

A thorough preoperative workup is essential before revision surgery. Ruling out periprosthetic joint infection involves laboratory testing such as erythrocyte sedimentation rate (ESR) and C-reactive protein (CRP), followed by joint aspiration for cell count, differential, and microbiology when indicated. Imaging, including plain radiographs and, in complex cases, preoperative CT scans, can assist in classifying bone defects and surgical planning. Multidisciplinary input is often required, involving infectious disease specialists for infection management, or plastic surgery for soft tissue coverage in cases with poor skin integrity. These steps are critical for optimizing surgical outcomes and should be understood by the broader healthcare team involved in patient care.

To sum up, revision knee arthroplasty is an essential but complex procedure for failed knee replacements. Understanding its indications, revision strategies (one-stage vs. two-stage), and specialised implant options enables multidisciplinary healthcare teams to support perioperative care, anticipate rehabilitation needs, and contribute effectively to patient education and recovery planning (see Table 3).

**Table 3:** Summary of revision knee arthroplasty types, strategies, and implant options

Category	Subtypes/options	Key features and clinical relevance
Revision strategies	One-stage revision	- Removal of failed components and reimplantation of new prostheses within the same procedure. - Suitable for aseptic loosening or low-virulence infections after thorough debridement [17].
	Two-stage revision	- First stage: implant removal, extensive debridement, antibiotic-loaded cement spacer placement. - Second stage: reimplantation of new components after infection eradication (usually six to 12 weeks). - Gold standard for infected revisions due to higher eradication rates [17].
Implant options	Constrained condylar knee (CCK)	- Offers enhanced coronal and sagittal plane stability. - Used when collateral ligaments are insufficient but a hinged implant is not required [18].
	Hinged implants	- Provides full mechanical constraint through a hinged mechanism between femoral and tibial components. - Indicated in severe ligamentous insufficiency, massive bone loss, or tumour resections [18].
	Stem extensions	- Long stems attached to femoral or tibial components to bypass bone defects and achieve stable diaphyseal fixation. - Enhances implant stability in compromised bone stock [18].
	Augments	- Metal wedges, porous metaphyseal sleeves, tantalum cones or blocks used to fill bone defects and restore joint line height and mechanical alignment. - Commonly used in tibial plateau or distal femur defects [18].
	Megaprostheses	- Extensive modular prostheses replacing large segments of bone. - Reserved for cases with severe bone loss where standard revision components are insufficient, often in oncology or salvage revisions [18].
Clinical considerations	-	- Revision TKA procedures are technically demanding with longer operative times and higher risks of infection, stiffness, and neurovascular complications. - Functional outcomes are generally lower than primary TKA, but meaningful pain relief and mobility improvements are achievable [4,18].

Discussion, limitations, and future implications

This review categorises knee arthroplasty into total, partial, and revision procedures, providing a structured educational framework for multidisciplinary healthcare teams. TKA remains the standard for advanced arthritis but carries risks such as infection, stiffness, and alignment challenges. PKA offers quicker recovery and more natural kinematics in selected patients but has higher revision rates due to disease progression in other compartments. Revision knee arthroplasty is complex, with higher complication rates and lower functional outcomes compared to primary procedures.

However, this study has limitations. As a narrative review, it does not follow a systematic methodology for literature selection, which introduces a potential for selection bias. The sources referenced were selected for their educational clarity and relevance rather than exhaustive coverage. In addition, the scope of the review prioritised breadth and foundational understanding over deep appraisal of outcomes, and some emerging technologies and long-term clinical data were intentionally omitted to maintain focus on practical classification for a multidisciplinary audience.

Future directions include validating this framework in educational settings to assess its effectiveness in improving knowledge and care delivery. Further research into implant design, alignment strategies, and robotic technologies is also essential to optimise surgical outcomes and enhance patient quality of life following knee arthroplasty.

## Conclusions

A comprehensive understanding of the various types and subtypes of knee arthroplasty-including total, partial, and revision procedures-is critical for multidisciplinary healthcare teams involved in perioperative and rehabilitative care. Each arthroplasty subtype carries distinct indications, surgical approaches, rehabilitation considerations, and complication profiles that influence patient outcomes. By utilising a structured educational framework, healthcare professionals can better navigate these complexities, enhance interprofessional communication, and contribute to more cohesive, patient-centred care.

Incorporating this framework into clinical training and routine practice has the potential to strengthen team-based decision-making, optimise rehabilitation strategies, and ultimately improve patient satisfaction and functional outcomes following knee arthroplasty.
